# Determination of endometrial cancer molecular subtypes using a whole exome-sequencing based single-method approach

**DOI:** 10.1007/s00432-024-05901-4

**Published:** 2024-07-25

**Authors:** Alexander Mustea, Damian J. Ralser, Eva K. Egger, Ulrike Ziehm, Sonia Vivas, Stephan Brock, David Jackson, Mateja Condic, Marc-A. Rauschendorf, Patrick Würfel, Frank Dombrowski, Lucia A. Otten, Pengming Sun, Anna Laib, Miguel Cubas Cordova, Rahel Hartmann, Martin A. Stein, Dominique Koensgen, Matthias B. Stope

**Affiliations:** 1 Department of Gynecology and Gynecological Oncology, University Hospital Bonn, Venusberg-Campus 1, Bonn, 53127 Germany; 2 Molecular Health, Kurfuersten-Anlage 21, Heidelberg, 69115 Germany; 3 Department of Surgery, DRK Kliniken Berlin Köpenick, Berlin, Germany; 4 Institute of Pathology, University Medicine Greifswald, Greifswald, Germany; 5 Laboratory of Gynecologic Oncology, Department of Gynecology, Fujian Maternity and Child Health Hospital, Affiliated Hospital of Fujian Medical University, No, 18 Daoshan Road, Fuzhou, 350001 China; 6 Dr. Senckenberg Institute of Pathology, University Hospital Frankfurt, Frankfurt am Main, Germany

**Keywords:** Endometrial cancer, Next generation-sequencing diagnostics, TCGA, Molecular subtypes, Molecular surrogate markers

## Abstract

**Aim:**

Endometrial cancer (EC) is heterogeneous with respect to epidemiology, clinical course, histopathology and tumor biology. Recently, The Cancer Genome Atlas (TCGA) network has identified four molecular subtypes with distinct clinical courses by an integrated multi-omics approach. These subtypes are of critical importance in the clinical management of EC. However, determination of TCGA molecular subtypes requires a complex methodological approach that is resource intensive and difficult to implement in diagnostic routine procedures. In this context, Talhouk et al. reported the precise determination of modified subtypes based on molecular surrogates obtained by a two-method approach comprising immunohistochemistry and DNA-sequence analysis (Proactive Molecular Risk Classifier for Endometrial Cancer; ProMisE). In this study, we aimed to identify EC molecular subtypes in analogy to TCGA and ProMisE applying an innovative whole exome-sequencing (WES) based single-method approach.

**Methods:**

WES was performed in a cohort comprising *N* = 114 EC patients. WES data were analyzed using the oncology treatment decision support software MH Guide (Molecular Health, Heidelberg, Germany) and EC molecular subtypes in analogy to TCGA and ProMisE were determined. Results from both classifications were compared regarding their prognostic values using overall survival and progression-free survival analyses.

**Results:**

Applying a single-method WES-approach, EC molecular subtypes analogue to TCGA and ProMisE were identified in the study cohort. The surrogate marker-analogue classification precisely identified high-risk and low-risk EC, whereas the TCGA-analogue classification failed to obtain significant prognostic values in this regard.

**Conclusion:**

Our data demonstrate that determination of EC molecular subtypes analogue to TCGA and ProMisE is feasible by using a single-method WES approach. Within our EC cohort, prognostic implications were only reliably provided by applying the surrogate marker-analogue approach. Designation of molecular subtypes in EC will be increasingly important in routine clinical practice. Thus, the single-method WES approach provides an important simple tool to tailor therapeutic decisions in EC.

## Introduction

Endometrial cancer (EC) is a heterogeneous malignancy concerning epidemiology, clinical course, histopathology and molecular tumor biology (Gaber et al. 2016; Brinton et al. 2013; Bokhman 1983; Cosgrove et al. 2018; Murali et al. 2018; Zannoni et al. 2010).

In 2013, The Cancer Genome Atlas (TCGA) Network reported a comprehensive genomic, transcriptomic and proteomic characterization of *N* = 373 EC samples applying a multi-omics approach (Cancer Genome Atlas Research Network et al. 2013). Based on these data, a classification of EC into four distinct and prognostically significant subgroups was deduced: (i) Polymerase Epsilon ultramutated (POLE), (ii) microsatellite instability hypermutated (MSI), (iii) copy-number (CN) low, and (iv) CN high EC. These 4 subtypes are characterized by distinct clinical outcomes. While EC patients with POLE mutation exhibit an excellent prognosis (Cosgrove et al. 2018; McAlpine et al. 2018; Stelloo et al. 2016; Karnezis et al. 2017; Bosse et al. 2018), EC patients classified as CN high display a limited prognosis. Patients who are classified as CN low and MSI-H exhibit an intermediate prognosis (McAlpine et al. 2018; Stelloo et al. 2017; Urick and Bell 2019). Of particular note, these distinct molecular subtypes are independent of histopathology highlighting genetic heterogeneity even within the same histological subgroup. From a clinical point of view, the prognostic value of these four molecular EC subtypes with distinct clinical courses is of highest relevance for treatment decision-making. However, characterization of patient-specific prognostic criteria based on the TCGA classification requires a complex methodological multi-omics approach that is resource intensive and difficult to implement in diagnostic routine procedures (McAlpine et al. 2018). In this context, Talhouk et al. reported the precise determination of modified four molecular subtypes, namely POLE, dMMR (deficient mismatch repair), p53 abnormal (p53abn), and no specific molecular profile (NSMP), with significant prognostic values based on molecular surrogates obtained by a two-method approach comprising immunohistochemistry and DNA-sequence analysis: (i) POLE status was determined by *POLE* DNA-sequence analysis, and (ii) MMR status (proficient/deficient MMR; (dMMR/pMMR) was assessed by MMR immunohistochemistry. p53 immunohistochemistry was applied as a surrogate for (iii) p53abn, and (iv) NSMP (Talhouk et al. 2015), resembling the TCGA CN high and CN low molecular subtype, respectively. This approach is referred to as ‘Proactive Molecular Risk Classifier for Endometrial Cancer’ (ProMisE). Of note, ProMisE was applied for the molecular classification analysis of the PORTEC-3 study cohort (León-Castillo et al. 2020). Thus, the prognostic significance of these molecular surrogates was confirmed in a prospective cohort. Currently, the ProMisE approach is applied as a routine diagnostic tool for the determination of EC molecular subtypes as suggested by national and international guidelines (Emons et al. 2023; Oaknin et al. 2022; Concin et al. 2021).

In the present study, we aimed to identify EC molecular subtypes in analogy to ProMisE and TCGA in a cohort comprising *N* = 114 EC patients, applying an whole exome-sequencing (WES) based single-method approach.

## Materials and methods

### Patient material

EC-cohort: Formalin-fixed and paraffin-embedded (FFPE) tissue of *N* = 114 patients diagnosed with EC between 2008 and 2016 were obtained from the pathological tumor tissue bank of the Greifswald University Hospital (Germany). The study was approved by the Ethics Committee of the Medical Faculty of the University of Greifswald (BB 121/17) and each patient provided written informed consent in accordance with the declaration of Helsinki principles. Baseline patient characteristics are depicted in Table 1.

**Table 1:** Clinicopathological characteristics of the Greifswald EC-cohort

	# (%)
**PFS events**	28
Missing (time or status)	1
**OS events**	20
Missing (time or status)	8
	months (range)
Median Follow up	59.5 (0-140)
	years (range)
**Median age (IQR)**	67.5 (58.8–73.3)
**Median BMI (IQR)**	31.2 (25.9–36.4)
	#
Missing (BMI)	6
**Stage (FIGO)**	# (%)
Stage 1	88 (81.4)
Stage 2	7 (6.5)
Stage 3	11 (10.2)
Stage 4	2 (1.9)
**Grade**	# (%)
G1	41 (38.0
G2	47 (43.5)
G3	20 (18.5)
**Histology subtype**	# (%)
Endometrioid	91 (84.3)
Mixed	6 (5.5)
Serous	2 (1.8)
Uterine carcinosarcomas	5 (4.6)
Clear cell	3 (2.8)
Adenocarcinoma with unknown histopathology	1 (1.0)
**Bokhman-type**	# (%)
Type I	91 (84.3)
Type II	17 (15.7)

*Note PFS* progression free survival, *OS* overall survival, *IQR* interquartile range, *BMI* body mass index

### Preparation of genomic DNA

Genomic DNA was extracted from FFPE EC tissue and normal adjacent tissue (NAT) using the QIAamp DNA FFPE Tissue Kit (Qiagen, Hilden, Germany) according to the manufacturer’s instructions. Briefly, FFPE tissue was lysed, and genomic DNA was bound to silica gel columns, washed, and eluted. A proteinase K digestion was performed to remove concomitant proteins. DNA samples were stored at -20 °C and shipped on dry ice.

### Whole exome-sequencing

Whole exome-sequencing (WES) of *N* = 114 EC and NAT samples was performed using a next generation sequencing (NGS) platform (Agilent SureSelect Human All Exon V6, Illumina HiSeq, SBS Kit v4, 2 × 100 bp read length) in an ISO/IEC 17,025 certified laboratory.

### MMR immunohistochemistry

MSI status based on WES data provided by MH Guide was compared to MSI-status obtained by MMR immunohistochemistry (IHC; MLH1, MSH2, MSH6, PMS2) for *N* = 59 cases from the EC-cohort.

### MH guide analysis

Read files (fastq format) from the Greifswald EC-cohort were analyzed using the oncology treatment decision support software MH Guide (version 4.1.2, Molecular Health, Germany), which is a system that interrogates NGS data to compile a list of reportable genetic variants and provides a summary of potentially effective medications, potentially ineffective medications, and medications that may come along with a higher risk of adverse reactions. All data collated are generated from an interactive database of curated, peer-reviewed and published evidence. MH Guide generates individual case reports which are downloadable as PDFs compiling all case relevant information including genetic-, biomarker-, detailed medical treatment options. MH Guide provides variant annotation as well as clinical interpretation of single-nucleotide variants (SNV), short insertion/deletion polymorphism (indels), somatic CN alterations, and MSI status. The MH Guide software is registered in Europe as an in-vitro diagnostic (IVD) medical device according to the latest EU regulation 2017/746 (IVDR) to support personalized cancer therapy. Through the IVDR status, it is subject to strict regulation, monitoring and surveillance. In the USA, Molecular Health is also certified by Centers of Medicare and Medicaid Services (CMS), under the Clinical Laboratory Improvement Amendments (CLIA), and is accredited by the College of American Pathologists (CAP) as a bioinformatics dry lab.

### Evaluation of EC molecular subtypes in analogy to TCGA

To classify the POLE group, somatic SNV that met the standard filtering criteria of MH Guide for paired whole exome analyses were used. Tumors with > 500 SNV, a CA rate of > 0.2, and a CG rate of < 0.03 were classified as POLE. For classification of MSS/MSI, the MSI detection tool MANTIS (Microsatellite Analysis for Normal-Tumor InStability; (Kautto et al. 2017) was used. If the stepwise difference is ≥ 0.3, an MSI-H biomarker is automatically added to the variants list. This threshold of 0.3 is based on validation of *N* = 40 TCGA cases from three cancer entities. MSI identification was validated with a limited number of samples that indicate 100% precision and recall between 60% and 100%. After identification of POLE and MSI classified EC, remaining specimen were further classified in CN high and CN low. To reproduce the CN high class according to the TCGA classification, we trained a supervised classification model to predict the CN cluster 4 of TCGA from the WES-based copy number calls of MH Guide. Cross-validation accuracy, precision, recall, and f1 were used to evaluate how well the classification based on DNA NGS data reproduced the original three platform molecular subtypes of TCGA.

### CN cluster prediction

CN clusters were extracted from the supplementary table datafile.S1.1.KeyClinicalData.xls (Cancer Genome Atlas Research Network et al. 2013) and binarized into 1 if a case belonged to cluster 4 and 0 if a case belonged to cluster 1, cluster 2, or cluster 3. 26% of samples fell into the CN cluster 4 class and 74% fell into the other classes. Multiple supervised classification algorithms and feature sets were tested with 5-fold cross-validation. 240 samples with available CN clusters were used for model training, and classification performance was evaluated by stratified 5-fold cross validation. Models were implemented in Python 3.6.8 using package scikit-learn 0.22.1.

A Naïve Bayes classifier with the following features was selected: Number of CN gains/CN losses per sample, number of CN gains/CN losses per chromosome, number of CN gains/CN losses per gene for the 25 most abundant genes, and ploidy and length of CN alterations per mega base. Although our features violate the assumption of uncorrelated features for a Naïve Bayes classifier, this model showed robust performance in predicting CN cluster 4 with average cross-validation accuracy, precision, recall, f1-sore, and ROC-AUC of 91.25%, 83.51%, 85.77%, 83.98%, and 95.08%, respectively, for the 240 training samples.

The CN cluster prediction algorithm was applied to samples from the Greifswald EC-cohort.

### Evaluation of EC molecular subtypes in analogy to ProMisE

To assess the feasibility of using surrogate markers for determination of molecular subtypes, as proposed by Talhouk et al. (Talhouk et al. 2015) by WES, patients of the Greifswald EC-cohort were stratified as follows: If a pathogenic or likely pathogenic mutation was found in *POLE*, EC were classified as POLE. Non-POLE tumors were further classified as dMMR and pMMR as described above. pMMR tumors were further assigned as p53abn if they had a somatic non-silent mutation in *TP53*. The remaining tumors were classified as NSMP, having no specific molecular pattern.

### Evaluation of risk stratification

The prognostic value of risk stratification analogue to the TCGA-classification and molecular surrogates was evaluated for the Greifswald EC-cohort using Cox proportional hazards regression and multivariable log-rank tests. Survival analysis was conducted in Python 3.6.8 using the package lifelines (0.23.9) and the survival package from R version 3.6.1.

## Results

*N* = 114 samples from the EC-cohort were included in the study. Sequencing was successful in *N* = 108 samples. For *N* = 7 samples, sequencing failed due to poor DNA quality. Median follow-up time in this cohort was 59.5 months (range 0-140 months). *N* = 91 (84,3%) EC displayed endometrioid histology (Type I EC), and *N* = 17 (15.7%) represented further histological subtypes (Type II EC), including serous (*N* = 2), clear cell (*N* = 3), carcinosarcomatous (*N* = 5) and mixed histology (*N* = 6). Clinicopathological characteristics are depicted in Table 1.

To determine EC molecular subtypes in analogy to both, TCGA-classification and molecular surrogates-classification (ProMisE), a single-method approach (MH Guide) was applied to WES data obtained from *N* = 101 cases of the EC-cohort. In analogy to TCGA, *N* = 9 (8.3%) cases were classified as POLE, *N* = 15 (13.9%) as CN high, *N* = 51 as MSI-H, and *N* = 33 as CN low. In analogy to the ProMisE surrogate marker approach, *N* = 6 (5,6%) cases were classified as POLE, *N* = 11 (10,2%) as p53abn, *N* = 53 as dMMR (49,1%), and *N* = 11 (10,2%) as NSMP (Table 2).

**Table 2:** Distribution of molecular subtypes in the Greifswald EC-cohort based on the MH guide approach for both classification systems, TCGA-analogue, and surrogate marker-analogue

TCGA-analogue			Surrogate marker-analogue		
Subtype	#	%	Subtype	#	%
POLE	9	8.3	POLE	6	5.6
MSI	51	47.2	dMMR	53	49.1
CN low	33	30.6	NSMP	38	35.2
CN high	15	13.9	p53abn	11	10.2

**Fig. 1:**
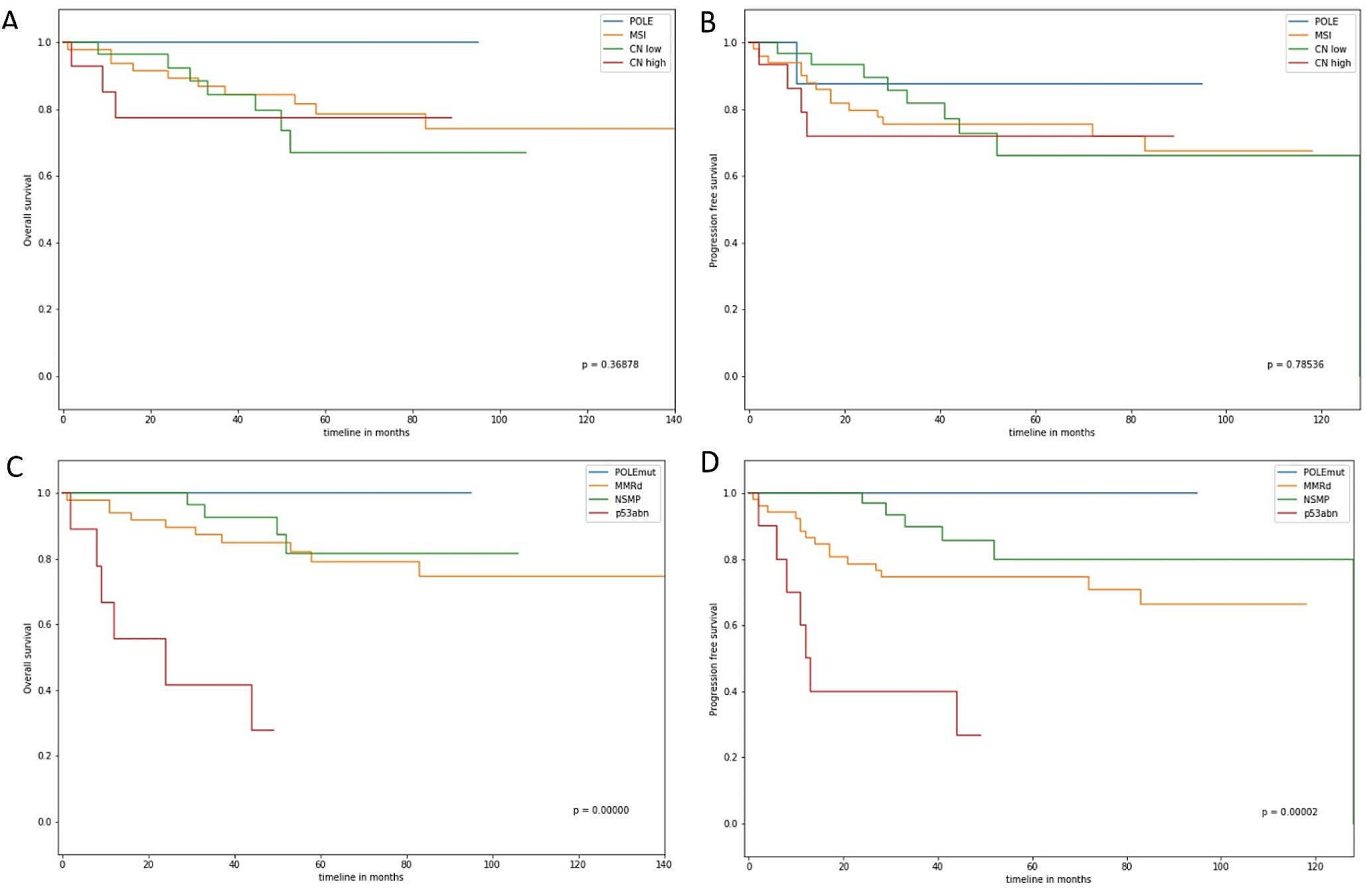
Kaplan-Meier survival curves for overall survival (OS; **A**, **C**) and progression-free survival (PFS; **B**, **D**) for the Greifswald EC-cohort. OS and PFS are shown for each molecular subtype in analogy to TCGA (**A**, **B**) and surrogate marker (ProMisE) classification (**C**, **D**)

In the EC-cohort, no correlation of molecular subtypes with Type I/Type II EC or histological subtypes was observed (Table 3). TCGA-analogue analysis classified one type II EC (clear cell histology) as POLE and only *N* = 4 type II EC were assigned as CN high. Following surrogate marker-based classification, one type I tumor was determined as POLE. *N* = 5 type II EC were assigned as p53abn. According to both classification systems, almost 50% of type II EC were classified as MSI/dMMR (Table 3). This proportion of MSI/dMMR EC, however, is greater compared to the published data (Cancer Genome Atlas Research Network et al. 2013; Talhouk et al. 2015; León-Castillo et al. 2020). To validate the ability of both classification systems, to identify low-risk and high-risk patients, Cox proportional hazard regression analysis was performed. Hazard ratios for POLE, MSI, CN high and CN low subtypes (TCGA-analogue classification) and hazard rations for POLE, dMMR, p53abn, and NSMP (surrogate marker-based classification) were obtained. The surrogate marker-based classification was superior compared to the TCGA classification in this cohort, particularly in identifying high-risk patients (Fig. 1). Stratification based on TCGA successfully identified the POLE and MSI groups but failed to identify the high-risk cases (hazard ratios for OS and PFS 1.2 and 1.3, respectively, with confidence intervals including 1; Table 4). Cox regression analysis confirmed this observation: For the surrogate marker-based classification, the log-rank test was highly significant (OS: *p* = 0.00056, PFS: *p* = 0.00067), and the hazard ratios for POLE and p53abn versus NSMP groups were very low and high, respectively, for both, OS and PFS (Table 4). As indicated, the proportion of MSI/dMMR EC was higher compared to the literature. To validate the robustness of MSI/dMMR determination based on WES data, MMR-IHC was performed for *N* = 59 patients. In this sub-cohort, *N* = 20 (33,9%) and *N* = 13 (22,7%) EC were classified as MSI/dMMR based on IHC and WES, respectively. However, the percentage of MSI/dMMR EC was significantly lower in this subgroup compared the overall cohort, implying a high rate of false positive MSI/dMMR tumors. However, the analyses show congruence of the data obtained from IHC and NGS in 88% of the cases.

**Table 3:** Clinicopathologic features of molecular subtypes presented separately for TCGA-analogue and surrogate marker-analogue classification, respectively

	TCGA-analogue				Surrogate marker-analogue			
	POLE	MSI	CN low	CN high	POLE	dMMR	NSM*P*	p53ab*n*
**Grading**								
1	4	12	18	7	3	13	23	2
2	4	28	12	3	3	28	13	3
3	1	11	3	5	0	12	2	6
**Stage (FIGO)**								
1	9	42	26	11	6	44	32	6
2	0	2	4	1	0	2	4	1
3	0	6	2	3	0	6	2	3
4	0	1	1	0	0	1	0	1
**Histology subtype**								
Adenocarcinoma (unknwon histopathology)	0	1	0	0	0	1	0	0
Endometrioid	8	43	29	11	6	44	35	6
Mixed	0	3	2	1	0	3	2	1
Clear cell	1	1	0	1	0	2	0	1
Uterine carcinosarcoma	0	2	1	2	0	2	1	2
Serous/serous-papillary	0	1	1	0	0	1	0	1
**Bokhman-type**								
Type I	8	43	29	11	6	44	35	6
Type II	1	8	4	4	0	9	3	5

**Table 4:** Log-rank p-values and hazard ratios for the predicted subtypes (TCGA-analogue and surrogate marker-analogue) for overall survival (OS) and progression-free survival (PFS) for the Greifswald EC-cohort with complete PFS/OS data. *number (%) of cases per subtype with complete OS/PFS survival. For POLE, hazard ratios are not reliable due to the small sample size

	Stratification scheme	Logrank pvalue	Predicted subtype	*n* (%)	Hazard ratio	Hazard ratio lower 95 CI	Hazard ratio upper 95 CI
OS (100)	TCGA-analogue	0.17337	POLE	9 (9.0)	0.000	0.000	Inf
			MSI	47 (47.0)	0.707	0.266	1.883
			CN high	14 (14.9)	1.205	0.310	4.676
	Surrogate marker-analogue	0.00056	POLEmut	6 (6.9)	0.000	0.000	Inf
			dMMR	49 (49.0)	1.441	0.447	4.646
			p53abn	10 (10.0)	12.840	3.460	47.646
PFS (107)	TCGA-analogue	0.74156	POLE	9 (8.4)	0.429	0.054	3.435
			MSI	50 (46.7)	1.013	0.422	2.435
			CN high	15 (14.0)	1.319	0.397	4.387
	Surrogate marker-analogue	0.00067	POLEmut	6 (5.6)	0.000	0.000	Inf
			dMMR	52 (48.6)	2.073	0.749	5.739
			p53abn	11 (10.3)	9.576	2.999	30.575

## Discussion

In modern oncology, molecular diagnostics are becoming increasingly important, particularly when it comes to individualized therapy decisions (Malone et al. 2020; Prasad et al. 2016). The incorporation of molecular markers into therapy stratification is intended to improve the treatment response and clinical outcome and to increase the safety of oncological therapy by reducing therapy-related toxicity (Murali et al. 2018; Urick and Bell 2019; Arend et al. 2018). The TCGA molecular subtypes of endometrial cancer, namely POLE, MSI, CN low, and CN high, and analogue classifications, in particular the ProMisE algorithm, are firmly established in clinical routine and are recommended for consideration in treatment decisions by national and international guidelines (Emons et al. 2023; Oaknin et al. 2022; Concin et al. 2021). Patients with POLE EC, typically displaying high-grade endometroid carcinomas, exhibit an excellent prognosis. Considering this excellent prognosis, overtreatment by means of adjuvant chemotherapy and/or radiation can be avoided. In contrast, the CN high subgroup comprises EC with predominantly serous-like histology, exhibit a very poor prognosis necessitating an extended therapy approach. The prognostic value of these retrospectively obtained four molecular subtypes was validated in a large prospective EC cohort (León-Castillo et al. 2020). From a clinical point of view, it is critically important to identify these different subtypes to avoid undertreatment in high-risk patients and overtreated in low-risk patients. To achieve this in clinical routine, a suitable diagnostic tool is crucial. Such a diagnostic tool is required to provide (i) a precise classification with high prognostic values and, (ii) should be methodologically feasible. The TCGA original publication is based on an extremely complex methodology comprising genomic, transcriptomic, and proteomic data (Cancer Genome Atlas Research Network et al. 2013). Talhouk et al. reported molecular surrogate markers obtained from immunohistochemistry and DNA sequencing to precisely identify four EC molecular subtypes with analogue prognostic values to the TCGA subtypes (ProMisE; (Talhouk et al. 2015; León-Castillo et al. 2020). In the present study, we investigated the feasibility of determining EC molecular subtypes in analogy to both, the TCGA- and molecular surrogates classification applying a WES-based single method approach that has been recently published (Mustea et al. 2023). Determination of EC molecular subtypes in analogy to TCGA-classification applying the single method approach has not yet been able to address high-risk patients with sufficient certainty. This could be due to methodological reasons. The DNA quality extracted from FFPE tissues in the EC-cohort was very heterogeneous. This might also be reflected by the comparatively high rate of MSI/dMMR cases (Hwang et al. 2017; Pécriaux et al. 2021). DNA quality analysis exhibited a very high proportion of short nucleic acid fragments, which may have led to the supposed detection of MSI/dMMR. Consistently, an increased number of MSI/dMMR tumors reduces the group of high-risk carcinomas. Comparable conclusions can be extrapolated regarding the CN status. Here, poor DNA quality may have also led to a decreased detection of CN high tumors. In this regard, we further investigated whether there is a correlation between MSI/dMMR status determined by MH Guide analysis and selected quality parameters. We found a strong correlation between MSI status and the fraction of mapped and on-target reads, the fraction of short fragments, and the amount of adapter contamination per sample (data not shown). EC samples were defined as MSI/dMMR if they had any of the following characteristics: Percentage of mapped reads < 92%, percentage of on-target reads < 66%, percentage of short fragments > 93%, or adapter contamination > 6.34%. All four parameters demonstrated a strong correlation with the age of the samples. All samples collected from 2011 onwards were within the non-critical range of parameter values. The default thresholds at which MH Guide triggers quality warnings for the fraction of mapped and on-target reads or short fragments are far more stringent than the critical values for these parameters determined by MSI definitions. It remains speculative whether the analysis would have been different with higher DNA quality. However, this has to be clarified conclusively in further investigations. The surrogate marker-based stratification by DNA sequencing, however, reliably identified low- and high-risk patients in the EC-cohort. The surrogate marker-based classification differs from the TCGA-analogue classification mainly with respect to the determination of the CN high/low or p53abn/NSMP group. Our data imply that based on p53 mutation status, at least in our cohort and in the presence of poor DNA quality, a stratification into high- or low-risk patients can be more reliably determined. Of note, *TP53* mutations were identified in all four subgroups, but did not show prognostic values in POLE and MSI/dMMR EC. This is clinically highly relevant and testing for all four subtypes should always be performed in routine clinical practice. If due to the complexity of the diagnostic procedure, testing for POLE is omitted, a p53abn but undetected POLE positive EC may be considered as high-risk with corresponding overtreatment.

Our data strongly support the inclusion of molecular subtyping in the diagnostic regimen of EC. Further, conduction of interventional trials performing stratification based on molecular subtypes is crucial and currently ongoing (e.g. RAINBO trial; NCT05255653; (RAINBO Research Consortium 2022). Our data further indicate, that identification of molecular subtypes in EC with subsequent risk stratification is feasible based on molecular surrogates obtained by a single method approach provided by MH Guide. The implementation of such a diagnostic tool in routine clinical practice is crucial to ensure safer and more effective treatment of EC patients. Due to the growing understanding of tumor biology, molecular markers will become increasingly important in the future (Arend et al. 2018; Stope et al. 2016; Vermij et al. 2020). In this context, new genetic markers can be easily integrated into the WES-based method allowing rapid translation of data from research into clinical practice. Furthermore, tumor genetic characterization provides the potential to identify targeted therapies based on tumor genetic alterations. This is therapeutically of great value especially in advanced disease stages after passing through different lines of therapy.

## Data Availability

No datasets were generated or analysed during the current study.
